# What Is Wrong With This Wireless Motility Capsule?

**DOI:** 10.14309/crj.0000000000000768

**Published:** 2022-05-04

**Authors:** Liana Mosley, Asad Jehangir, Subbaramiah Sridhar, Amol Sharma, Satish S.C. Rao

**Affiliations:** 1 Medical College of Georgia at Augusta University, Augusta, GA

## CASE REPORT

**Figure 1.**
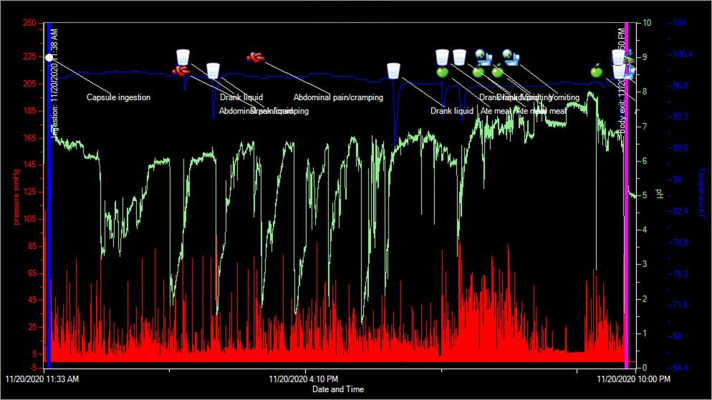
Wireless motility capsule profile of the patients. The horizontal axis shows the time scale, and the vertical axis shows pressure (red), pH (green), and temperature (blue). The pH was 5–7 with occasional decreases to 2.0 coinciding with drinks/meals, indicating esophageal capsule retention. Ten hours later, the patient vomited and expelled the capsule, confirmed by a temperature decline.

**Figure 2.**
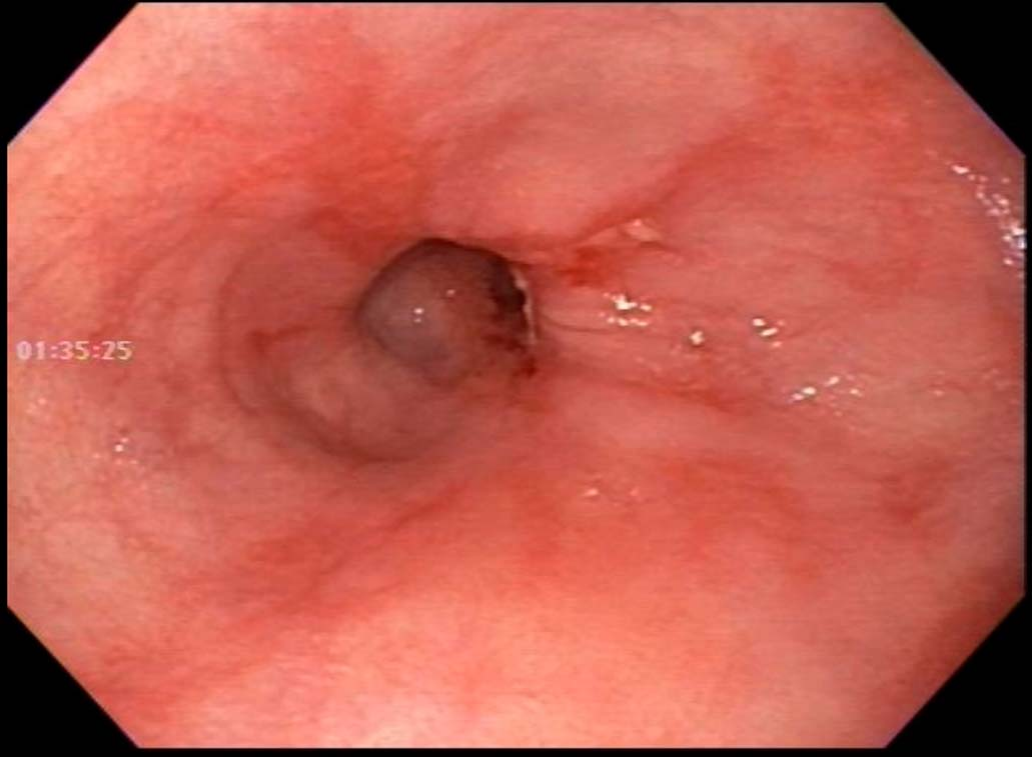
Upper endoscopy 1 week after wireless motility capsule testing confirming distal esophageal stricture with mucosal changes of esophagitis.

A 37-year-old woman was evaluated for suspected gastroparesis with nausea, heartburn, and constipation. She reported bowel movements every 2 weeks (Bristol Stool Scale Type I–III) with excessive straining. She was taking pantoprazole, famotidine, promethazine, and bisacodyl. Her history included esophageal stricture requiring dilation 3 years previously. She reported intermittent dysphagia for solids. A wireless motility capsule (WMC) study was performed for the evaluation of gastrointestinal motility disorder. The WMC profile is presented (Figure 1). The horizontal axis shows the time scale, and the vertical axis shows pressure (red), pH (green), and temperature (blue). The pH was 5–7 with occasional decreases to 2.0 coinciding with drinks/meals, indicating esophageal capsule retention. Ten hours later, the patient vomited and expelled the capsule, confirmed by a temperature decline. A week later, an upper endoscopy confirmed distal esophageal stricture with mucosal changes of esophagitis (Figure 1). Pantoprazole was increased to twice daily, and repeat esophagogastroduodenoscopy in 8 weeks was recommended (Figure 2).

The WMC study is contraindicated in patients with dysphagia, stricture, or bowel obstruction. Capsule retention, although rare (0.33%), can occur, especially if the contraindications for this test are not carefully ruled out before ordering the test.^1^ The test was incorrectly requested for the patient presented here, but fortunately, the patient vomited the capsule. In patients with dysphagia, stricture, or bowel obstruction, gastrointestinal dysmotility symptoms should be evaluated using alternative tests, such as scintigraphy. Although WMC is significant advance and clinically useful for the diagnosis of gastrointestinal motility disorders, the manufacturer's recommendations and precautions should be followed carefully to prevent potentially serious complications.^2^

## DISCLOSURES

Author contributions: L. Mosley wrote the manuscript. A. Jehangir edited the manuscript. S. Sridhar, A. Sharma, and S.S.C. Rao revised the manuscript for intellectual content. S.S.C. Rao is the article guarantor. All authors approve the final version of the manuscript.

Financial disclosure: None to report.

Previous presentation: This case was presented at the American College of Gastroenterology Annual Scientific Meeting; October 22–27, 2021; Las Vegas, Nevada.

Informed consent was obtained for this case report.
